# Supplementary material for: Impact of extent of internal acoustic meatus tumor removal using translabyrinthine approach for acoustic neuroma surgery
Source: PLoS One. 2021 Aug 5;16(8):e0253338. doi: 10.1371/journal.pone.0253338 (PMC8341598; doi:10.1371/journal.pone.0253338)
Supplement: S1 File — This is the supporting information of our included cases. (PDF) [file pone.0253338.s001.pdf]

PDF-XChange Product
Click to buy NOW!
www.tracker-software.com

ase	Sex	AGE	Duration	Post-OP facial	Tumor size	Tumor type :	IAC involvemer	Tumor removal,	IAC tumoval	Facial numbness	Lower cranial ne	Pre-OP hearing	Pre-OP GKRS	Post-OP GKRS	post-op CSF	distance
1	2	47	12	1	40	2	3	4	1	2	2	64	0	1	0	4.7
2	2	27	21	6	54	2	3	3	2	1	2	110	0	1	0	3.7
3	1	31	12	1	30	3	3	3	2	2	1	55	0	0	0	6.1
4	2	55	12	2	37	2	3	3	1	2	2	63	0	0	0	5.1
5	2	63	13	3	36	2	3	4	2	2	2	110	0	0	0	2.4
6	2	35	12	1	30	2	3	3	1	1	2	48	1	0	0	8.3
7	2	41	13	1	30	1	3	4	2	2	2	74	0	0	0	6.2
8	1	32	14	4	40	3	3	4	2	1	2	75	0	0	0	9.7
9	1	24	9	3	43	2	3	4	2	2	2	15	0	0	0	6.7
10	2	69	28	4	34	3	3	4	2	1	2	65	0	0	0	6.7
11	2	33	7	3	30	2	3	3	2	1	2	65	0	0	0	5.8
12	1	36	10	2	48	2	3	2	2	2	2	55	0	1	0	8.1
13	1	28	10	3	43	3	3	4	2	1	2	110	1	0	0	7.8
14	2	57	18	1	45	2	2	3	2	2	2	70	0	0	0	8.3
15	2	69	21	2	40	3	3	1	1	1	1	110	0	0	0	6.7
16	2	77	9	2	35	3	3	4	2	1	2	65	1	0	0	6
17	2	24	9	1	35	2	3	3	2	1	2	25	0	0	0	7
18	2	33	14	5	55	2	3	2	2	2	1	110	0	1	0	0
19	2	44	14	1	32	1	2	3	2	1	2	45	0	0	0	8.9
20	2	30	12	1	37	1	3	3	0	1	2	110	0	0	0	7
21	1	48	6	4	35	1	2	3	2	1	2	110	0	0	0	7.8
22	1	52	6	1	32	1	3	3	2	1	2	63	0	0	0	6.2
23	1	43	6	1	53	3	3	2	2	2	1	80	0	0	0	8.2
24	1	55	8	4	46	3	2	3	2	2	1	42	0	0	0	4.1
25	1	36	6	2	42	3	3	3	2	1	1	110	0	0	0	9.7
26	2	36	6	1	30	3	2	4	2	2	1	110	0	0	0	3.3
27	1	44	11	2	48	3	2	3	2	1	1	110	0	0	1	5.2
28	1	34	10	1	35	1	3	4	2	1	2	25	0	0	0	4.6
29	1	39	13	1	30	2	3	2	1	1	2	15	0	0	0	7.4
30	1	58	26	1	41	3	3	4	1	2	2	110	0	0	0	7.4
31	2	25	10	1	40	2	3	3	2	2	2	50	0	1	0	8.4
32	1	51	7	3	37	3	3	2	2	1	1	30	0	1	0	9.9
33	1	57	7	1	43	3	3	4	1	1	1	75	0	0	0	6.9
34	2	59	7	4	50	2	2	4	2	1	2	100	0	0	0	7.3
35	2	54	10	2	31	2	3	4	2	1	2	110	0	0	0	7.7
36	2	57	12	4	37	2	3	3	2	1	2	110	0	0	0	1.8
37	2	53	10	1	37	2	3	3	1	1	2	45	0	0	0	4.8
38	2	41	6	2	37	2	3	4	2	1	2	45	0	0	0	7.8
39	2	22	7	2	32	2	3	3	1	1	2	75	0	1	0	1
40	2	61	15	1	30	1	3	4	2	2	2	100	0	0	0	5.7
41	1	38	16	2	46	2	3	4	2	1	2	50	0	0	1	6.8
42	1	55	8	1	37	3	2	4	2	2	1	100	0	0	1	3.9
43	2	55	11	1	41	2	2	4	2	1	2	66	0	0	1	6.3
44	1	61	7	1	41	3	3	4	2	2	2	85	0	0	0	11.2
45	1	63	6	4	32	3	3	4	2	1	2	70	0	0	0	9.6
46	1	47	8	3	37	2	3	4	2	2	2	110	0	0	0	10.1

PDF-XChange Product
Click to buy NOW!
www.tracker-software.com

PDF-XChange Product
Click to buy NOW!
www.tracker-software.com

47	1	35	7	1	30	1	3	3	2	1	2	25	0	0	0	7.8
48	2	52	7	1	32	2	3	4	2	1	2	50	0	0	1	3.4
49	2	35	8	4	31	3	3	4	2	1	2	15	0	0	0	9.5
50	1	52	25	1	30	2	3	4	2	2	2	100	0	0	0	8.9
51	2	52	7	3	46	2	3	4	2	1	1	110	0	0	0	3.8
52	1	65	11	1	41	2	2	3	2	1	2	76	0	0	0	3.8
53	2	43	18	1	38	2	3	4	2	1	2	110	0	0	0	8.9
54	1	45	6	1	35	2	2	3	2	1	2	26	0	0	0	8.5
55	1	49	6	4	40	3	3	4	2	1	2	51	0	0	0	6.6
56	2	54	16	1	42	3	3	3	2	1	2	110	0	0	0	4.3
57	2	47	9	3	31	1	3	4	2	1	2	59	0	0	0	8.8
58	1	52	8	1	50	2	3	3	2	1	2	55	0	0	0	5.5
59	1	37	6	1	36	2	3	4	2	2	2	40	0	0	0	10.5
60	2	31	26	1	46	2	3	4	1	1	2	110	0	1	1	1
61	2	37	8	1	32	2	3	4	1	2	2	64	0	0	0	4.2
62	2	28	8	1	38	2	3	4	2	2	2	15	0	0	1	8.6
63	1	50	6	1	55	2	2	4	2	1	1	58	0	0	1	6.9
64	2	35	7	1	35	1	3	4	2	1	2	101	0	0	0	4.6
65	2	56	13	1	46	2	3	3	1	1	1	110	0	1	0	1.7
66	2	52	10	2	44	1	3	4	2	1	1	28	0	0	0	6.8
67	1	32	6	2	39	3	3	3	2	2	2	10	0	0	0	10.4
68	2	47	7	2	42	2	3	4	2	1	2	66	0	0	0	5
69	1	45	5	4	39	2	3	4	2	2	2	66	0	0	0	10.9
70	1	24	11	2	62	2	3	4	2	2	2	110	0	0	0	10.7
71	2	62	8	2	35	2	2	4	2	1	2	85	0	0	0	1.9
72	2	55	7	2	40	2	3	4	2	1	1	78	0	0	0	4.2
73	1	42	6	1	30	2	3	4	2	2	2	20	0	0	0	7.1
74	1	54	9	2	45	3	2	3	2	1	2	49	0	1	0	5.6
75	2	71	18	3	30	1	3	4	1	2	2	70	0	0	0	7.3
76	2	62	12	2	39	1	3	4	2	2	2	110	0	0	0	5
77	2	58	7	3	34	3	3	3	2	2	1	110	1	0	0	7.5
78	1	63	14	1	32	3	2	4	2	2	2	85	0	0	1	9.3
79	1	55	6	2	31	2	3	4	2	1	2	83	0	0	0	8.4
80	2	65	12	1	31	2	3	4	2	1	2	110	0	0	0	4.4
81	1	57	12	1	39	3	2	3	2	2	2	110	0	0	0	5
82	1	48	5	1	46	3	3	4	2	2	2	49	0	0	0	7.4
83	2	57	8	1	40	2	3	4	2	1	2	49	0	0	0	7.6
84	2	40	5	1	39	2	3	3	2	1	2	41	0	0	0	7.5
85	2	25	7	1	47	1	3	3	2	1	2	58	0	0	0	10.1
86	1	48	6	1	38	3	3	4	2	2	2	26	0	0	0	9.7
87	1	29	6	2	34	1	3	3	2	2	2	61	0	0	0	8.6
88	1	67	8	2	36	1	3	3	2	2	2	93	0	0	0	5.4
89	1	57	6	1	44	2	2	3	2	1	2	110	0	0	0	5
90	2	52	6	2	39	1	1	2	0	2	2	20	0	0	0	6.4
91	2	34	9	2	56	2	3	3	2	1	2	28	0	0	0	1.4
92	2	68	6	4	42	2	3	3	2	1	2	110	0	0	1	7

PDF-XChange Product
Click to buy NOW!
www.tracker-software.com

93	2	30	7	1	35	2	2	4	2	1	2	35	0	0	0	10.3
94	2	45	19	1	37	1	1	3	0	1	1	39	1	0	1	3.5
95	1	47	7	2	35	1	3	3	2	1	2	105	0	0	0	10.8
96	2	62	8	2	39	2	3	3	2	1	2	110	0	0	0	4.8
97	2	55	9	1	42	3	1	4	0	1	2	89	0	0	0	7.8
98	1	55	19	1	39	2	3	3	2	1	2	68	0	0	1	8.8
99	1	54	5	1	40	1	2	4	2	1	2	110	0	0	0	6.8
100	2	69	8	1	52	2	3	3	2	1	2	63	0	0	0	10.8
101	1	24	7	1	39	2	3	3	2	2	2	54	0	0	0	8.8
102	1	25	9	1	33	1	3	3	2	1	2	110	0	0	0	6.2
103	1	39	8	2	38	2	3	3	2	1	2	15	0	0	0	2.2
104	1	52	5	3	41	3	3	3	2	1	2	48	0	0	0	10.9
