# Supplementary material for: Impact of extent of internal acoustic meatus tumor removal using translabyrinthine approach for acoustic neuroma surgery
Source: PLoS One. 2021 Aug 5;16(8):e0253338. doi: 10.1371/journal.pone.0253338 (PMC8341598; doi:10.1371/journal.pone.0253338)
Supplement: Attachment — Submitted filename: Response to reviewers.docx [file pone.0253338.s002.docx]

# Journal Submissions Rebuttal Letter

DATE 1/29/2021

Dear Dr. Alyono,

Thank you for inviting us to submit a revised draft of our manuscript entitled "Impact of extent of internal acoustic meatus tumor removal using translabyrinthine approach for acoustic neuroma surgery" to PLOS ONE. We also appreciate the time and effort you and each of the reviewers have dedicated to providing generous comments on the manuscript to strengthen our paper. Thus, it is with great pleasure that we resubmit our article for further consideration. We have edited the manuscript to reflect the detailed suggestions you have graciously provided. We also hope that our edits and the responses we provide below satisfactorily address all the issues and concerns you and the reviewers have noted.

To facilitate your review of our revisions, the following is a point-by-point response to the questions and comments delivered in your letter.

**EDITOR SUGGESTIONS:**

1. *Statistically, there is likely a multicollinearity issue.  Extent of IAC removal is unlikely to be independent of extent of IAC involvement.  In addition, extent of IAC removal is unlikely to be independent of overall tumor removal. For example - might partial IAC involvement be more likely to be completely removed as opposed to a tumor completely filling the canal?*
   - **RESPONSE:** Thank you very much for your question. Yes, there might be collinearity issue between extent of IAC removal and IAC involvement. And there might also be collinearity issue between extent of IAC removal and overall tumor removal. However, we consider the issue may not exist for the following reasons. First, if there was multicollinearity issue among these three factors. When we put all these three factors in the regression model, we should find no statistics significance in all these three factors. On the other hand, we do see the statistics significance in the factor of extent of IAC removal which showed in table 2. Second, the percentage of facial palsy in partial IAC involvement and total ISC tumor removal was quite different (16% vs 26%) which was shown in revised table 2. Third, we do not see correlation or upward trend between the extent of overall tumor removal and percentage of facial palsy which was shown in revised table 2.

**REVIEWER 1 COMMENTS:**

1. *Line 59: suggest replacing “nonuseful” with “non-serviceable”*
   - **RESPONSE:** Thank you for this suggestion. We have corrected the words in our revised manuscript.
2. *Line 92: suggest replacing “facial palsy” with “facial function”*
   - **RESPONSE:** We have corrected the words in our revised manuscript. Thank you for help.
3. *Lines 99-104: Please provide a figure illustrating how the measurements were performed.*
   - **RESPONSE:** We illustrated the measurements of the minimal distance ( ) between the internal auditory canal and jugular bulb by one patient’s coronal view of a temporal bone CT scan as following.

IAC🡪

Jugular bulb

4. *Line 104: Please either provide a figure or describe the “previously reported scales”.*
   - **RESPONSE:** We cited with reference 4 and 8 for extent of tumor removal scales as following: The percentage of tumor removal was determined by serial MR imaging and surgical findings of surgeons. We defined the extent of tumor removal as follows: total removal represented no visible tumor remnant at the end of surgery and serial MR imaging; near total removal was any residual tumor ≤ 5%; subtotal removal when the tumor was resected 80% to 90% by volume; partial removal was tumor remnant more than definition of subtotal removal. We have added this comment to the section of study design. Thank you for this suggestion.
5. *Line 104-105: Please rephrase this sentence, it is not clear what the authors are trying to state.*
   - **RESPONSE:** Thank you for this suggestion. We have removed this sentence because that was redundant after careful consideration.
6. *Line 107: would not use the word “invasion” as this suggests a malignant process.*

- **RESPONSE:** Thank you for this suggestion. We have revised the word with “involvement” in our revised manuscript.

7. *Line 108: what do the authors mean by partial? What percentage of tumor removal would be categorized as “partial”?*
   - **RESPONSE:** We defined the extent of IAC tumor removal as none, partial, and total removal. If remnant tumor was found in inner auditory canal by MRI performed 3 days postoperatively, that would be classified to “partial”.
8. *Line 112: Were previously radiated patients included in the study?*
   - **RESPONSE:** Yes, we enrolled 5 cases previously radiated at other hospital but the tumor size was still larger than 3cm. We have recorded as “Pre-op radiosurgery” group at table 1.
9. *Line 113-115: Please re-word this sentence.*

- **RESPONSE:** Thank you for this suggestion. We have revised the sentence “Stereotactic radiotherapy or radiotherapy” to “gamma-knife radiosurgery (GKRS)” in our revised manuscript.

10. *Lines 117-118: Awkwardly worded sentence, please change.*
    - **RESPONSE:** Thank you for this suggestion. We have removed this sentence because that was redundant after careful consideration.
11. *Lines 125-126: Would remove this sentence.*

- **RESPONSE:** Thank you for this suggestion. We have removed this sentence in our revised manuscript.

12. *Lines 137: Please rephrase this sentence.*

- **RESPONSE:** Thank you for this suggestion. We have rephrased this sentence as “More than 87% patients had non-serviceable hearing in the diseased ear. Preoperative hearing level was assessed by pure tone audiometry (average threshold for 0.5, 1, 2, and 4 kHz) and showed a mean preoperative hearing level of 71.21 ± 31.94 dBHL” in our revised manuscript.

13. *Line 137-141: Please put a reference to the AAO HNS classification guidelines and either describe what the classes are or place a table showing what this is. Was there a 71 dB average drop in hearing? That would be surprising given that these are all translab approaches. If this it the case, I would recommend reporting the pre-op and post-op discrimination scores.*
    - **RESPONSE:** We are sorry for misunderstanding and the sentence was rephrased to “preoperative hearing level” instead of dropped hearing.
14. *Table 1:
    - Again, what is the difference between subtotal and partial tumor removal The authors do not make this clear.*

- **RESPONSE:** We cited with reference 4 and 8 for extent of tumor removal scales as following: The percentage of tumor removal was determined by serial MR imaging and surgical findings of surgeons. We defined the extent of tumor removal as follows: total removal represented no visible tumor remnant at the end of surgery and serial MR imaging; near total removal was any residual tumor ≤ 5%; subtotal removal when the tumor was resected 80% to 90% by volume; partial removal was tumor remnant more than definition of subtotal removal. We have added this comment to the section of study design. Thank you for this suggestion.

15. *- “Low” cranial nerve should be “Lower”*
    - **RESPONSE:** Thank you for this suggestion. We have corrected the word in our revised manuscript.
16. *Table 3:*

*- What does GKS stand for?*

- **RESPONSE:** We are sorry for misunderstanding. The proper word should be “gamma-knife radiosurgery (GKRS)”. We have corrected the word in our revised manuscript.

17. *Lines 155-157: The facial nerve stimulate on the nerve monitor at the end of the case?*
    - **RESPONSE:** Yes, we checked facial nerve at the end of the case.
18. *Lines 192-193: Please re phrase this sentence.*
    - **RESPONSE:** Thank you for this suggestion. We have removed this sentence in our revised manuscript.
19. *Lines 203-207: While the authors found a statistically worse facial nerve outcome in cases that involved the IAC, I would not call this a novel idea. This is inferring that more stretching and manipulation of the nerve leads to a worse facial nerve outcome, which isn’t surprising and does not necessarily need statistical tests to know.*
    - **RESPONSE:** We agreed that postoperative facial palsy might be influenced by manipulation of nerve during operation. In our manuscript, we also suggested that surgeons should take care when manipulating facial nerves while removing tumors in the internal acoustic meatus as well as in the cerebellopontine angle at line 197 to 199 (revised version). We have removed the word “novel” in our revised manuscript.
20. *Line 225: Would replace the word ‘demands’ in this sentence.*
    - **RESPONSE:** Thank you for this suggestion. We have rephrased the sentence as “According to our findings, the young patient group had more chance to receive post-operative GKRS (p = 0.027)” in our revised manuscript.

**REVIEWER 2 COMMENTS:**

1. *What was the mean duration of follow up (and the ranges) used to assess tumor control? Were any tumors observed beyond the three month post-op MRI, or was that the cutoff to assess tumor control? If not, do the authors feel confident that they are capturing all cases of subsequent tumor growth following such a short follow-up period? While the end results may not ultimately change, I would like to see a longer duration of follow-up before reaching the conclusions drawn in the study with regards to tumor control.*
   - **RESPONSE:** Thank you for providing these insights. The mean duration of follow up of 104 cases was 39 months, the ranges of duration were 6-107 months and the median duration was 34.5 months. We arranged periodic MRI scans within three days and three months after surgery to monitor tumor regrowth of the residual tumor. After then, we checked MRI every 6 to 12 months to assess tumor control. According to our study, the mean duration from operation to receiving GKRS of 10 cases was 12 months, the ranges of duration were 4 to 25 months. Therefore, it can be proved that our duration of follow-up time was enough for surveillance of tumor recurrence. We have supplemented the result with explanations of above data in our revised manuscript.
2. *Were any patients treated prophylactically with GKS prior to demonstrating tumor growth?*
   - **RESPONSE:** No, all of patients did not treat prophylactically with GKRS. Postoperative GKRS was arranged only when the size of tumor remnant enlarged.
3. *It appears that the 3 patients without IAC tumor involvement were included in the full analysis. Was there any consideration to exclude these patients? Why or why not? Should we suppose that IAC tumor involvement is a factor in facial nerve outcome or tumor control rate, or the manipulation (or lack thereof) within the IAC?*

- **RESPONSE:** Thank you for providing these insights. There were 3 patients without IAC tumor involvement who were included in the 4 patients without IAC tumor removal. That means no perioperative manipulation of IAC and facial nerve if patients without IAC tumor involvement. Therefore, extent of IAC tumor removal seems to be more significant factor of our surgical outcome.

4. *To follow the last point, can the authors please expand the data in Table 2 to reflect the number of patients in each subgroup with good facial nerve function?*
   - **RESPONSE:** Thank you for this suggestion. We have incorporated your comments by expanding the data in Table 2 in our revised manuscript.
5. *The authors state linear regression was used to determine the significance of various independent variables on facial nerve outcomes and tumor control. Did the authors consider any form of adjusted multivariate regression models to control for various confounding factors? In all tables with p-values, it is suggested that authors denote the type of statistical test used and include in footnotes all adjustments accounted for in the modeling framework, if used.*
   - **RESPONSE:** Thank you for this suggestion. We have revised the tables with the type of statistical test used in our revised manuscript.
6. *Were any patients treated with postoperative steroids? Did this have any impact on facial nerve outcomes?*
   - **RESPONSE:** You have raised an important point; however, more than 90% patients treated with postoperative steroids for prevention of postoperative brain edema and increased intracranial pressure. Because most of patients treated with postoperative steroids, we consider that would not have impact on our results.
7. *The authors report two patients with tumor involvement within the mastoid segment of the facial nerve. This would represent an unusual tumor growth pattern. Could the authors please expand on this further?*

- **RESPONSE:** Thank you for providing these insights. We rechecked the pathology report of these patients with tumor involvement within mastoid segment of the facial nerve that indeed proved as vestibular schwannoma. However, large tumor size of these two cases may have impact of extrusion on nerve. We applied the preoperative MRI from one of the cases for example as follows:

8. *The authors suggest that there are no prior studies regarding the impact of tumor remnant with facial nerve outcomes or recurrence. Did the authors consider the following: Bloch DC, Oghalai JS, Jackler RK, Osofsky M, Pitts LH. The fate of the tumor remnant after less-than-complete acoustic neuroma resection. Otolaryngol Head Neck Surg 2004;130:104-12? How does the present study compare to the findings from prior studies with regards to tumor control based on the degree of tumor removal?*
   - **RESPONSE:** We appreciate your comments and drew some comparison with this article. Compared to Bloch’s study, there were 44 cases with near-total resection and 7 cases with partial and subtotal resection in our study under the same definition of resection extent. In the group of near-total resection, the recurrence rate was 11.4% and lower than 42.9% of subtotal resection. Though our results seemed to be worse than Bloch that reported with 3% and 32% recurrence in near-total and subtotal resection separately, large tumor size of our cases may affect this outcome. However, the suppose of near-total resection having greater therapeutic outcome and a lower risk of recurrence was approved not only in previous studies but our results. Postoperative alert surveillance with serial MRI to detect early recurrence is necessary. We have added this comment to the section of discussion.
9. *The introduction seems non-linear, including some methodological details of the study regarding surgical approach, surgical teams, etc. Consider consolidating these details to the methods section for better flow.*
   - **RESPONSE:** Thank you for this suggestion. We have modified the methodological details from introduction to the methods section in our revised manuscript.
10. *Line 137: “Most” to “more”? “Healing” to “hearing”*
    - **RESPONSE:** We agreed with your comments and have corrected the words in our revised manuscript.
11. *Line 192-193: Can you please clarify this sentence – these patients were not suggested for what?*

- **RESPONSE:** Thank you for this suggestion. We have removed this sentence because that was redundant after careful consideration.

**CONCLUDING REMARKS**: Again, thank you for giving us the opportunity to strengthen our manuscript with your valuable comments and queries. We have worked hard to incorporate your feedback and hope that these revisions persuade you to accept our submission.

Sincerely,

***Mao-Che Wang***

Corresponding Author

Department of Otorhinolaryngology-Head and Neck Surgery, Taipei Veterans General Hospital, and School of Medicine, National Yang-Ming University, Taiwan

No. 201, Sec. 2, Shih-Pai Rd.

Taipei 11284, Taiwan.

Tel: +886 2 27857337 ext.151

Fax: + 886 2 28757338

E-mail: [wangmc@vghtpe.gov.tw](mailto:wangmc@vghtpe.gov.tw)
