# Supplementary material for: Impact of extent of internal acoustic meatus tumor removal using translabyrinthine approach for acoustic neuroma surgery
Source: PLoS One. 2021 Aug 5;16(8):e0253338. doi: 10.1371/journal.pone.0253338 (PMC8341598; doi:10.1371/journal.pone.0253338)
Supplement: Attachment — Submitted filename: Response to reviewers.docx [file pone.0253338.s003.docx]

# Journal Submissions Rebuttal Letter

DATE 3/31/2021

Dear Dr. Alyono,

Thank you for inviting us to submit a revised draft of our manuscript entitled "Impact of extent of internal acoustic meatus tumor removal using translabyrinthine approach for acoustic neuroma surgery" to PLOS ONE. We also appreciate the time and effort you and each of the reviewers have dedicated to providing generous comments on the manuscript to strengthen our paper. Thus, it is with great pleasure that we resubmit our article for further consideration. We have edited the manuscript to reflect the detailed suggestions you have graciously provided. We also hope that our edits and the responses we provide below satisfactorily address all the issues and concerns you and the reviewers have noted.

To facilitate your review of our revisions, the following is a point-by-point response to the questions and comments delivered in your letter.

**EDITOR SUGGESTIONS:**

1. *Regarding involvement of the mastoid segment: does this mean the mastoid segment of the facial nerve had vestibular schwannoma within it? How is this distinguished from facial nerve schwannoma? The pathology report alone cannot distinguish the nerve of origin.*
   - **RESPONSE:** Thank you very much for your question. All of our patients enrolled to our study had no preoperative facial palsy and diagnosed as huge tumor size >3 cm. That would be less possible for patients with huge size of facial nerve schwannoma who have no preoperative facial palsy in our experience.

**REVIEWER 1 COMMENTS:**

- Thank you for suggestion of further English Language proof-reading service. The manuscript has been carefully reviewed by an experienced editor whose first language is English again. We have corrected the grammatical errors in our revised manuscript.

2. *Lines 64-65: This sentence needs to be rephrased. There certainly are papers discussing IAC tumor removal. The authors need to make this sentence clearer.*
   - **RESPONSE:** Thank you for this suggestion. We have rephrased this sentence in our revised manuscript.
3. *Line 72: ‘Incidence’ should be ‘prevalence’.*
   - **RESPONSE:** We have corrected the word in our revised manuscript. Thank you for help.
4. *Lines 72-75: This sentence should be revised. Facial nerve palsy complicating surgery is awkwardly phrased.*
   - **RESPONSE:** Thank you for this suggestion. We have rephrased this sentence in our revised manuscript.
5. *Lines 86: Please consider removing this sentence. It is redundant, you have already stated the translab approach was used in the previous sentence.*
   - **RESPONSE:** Thank you for this suggestion. We have removed this sentence because that was redundant after careful consideration.
6. *Line 89-90: This sentence needs revision, either remove ‘and reports’ or change the sentence to avoid having two ‘and’s’.*
   - **RESPONSE:** We have removed the words ‘and reports’ in our revised manuscript. Thank you for help.
7. *Lines 107-109: Please add a figure showing how the measurements were made.*

- **RESPONSE:** We illustrated the measurement of tumor size using the maximal diameter of the extracanalicular component from the axial/coronal view of MRI scan as following.

8. *Line 114: ‘surgical findings of surgeons’ is awkward and redundant, should just be ‘intraoperative findings’*
   - **RESPONSE:** Thank you for this suggestion. We have rephrased this sentence in our revised manuscript.
9. *Line 125: ‘and other pathological types’ is vague and needs rewording*
   - **RESPONSE:** We have rephrased this sentence as “All patients aged <20 years, neurofibromatosis type II (NF2), non-vestibular schwannoma, recurrent tumor, preoperative facial palsy grade III–VI, and mortalities were excluded from the study.” in our revised manuscript. Thank you for help.
10. *Lines 127-130: Sentence needs to be rephrased. It is repetitive and awkwardly rephrased.*

- **RESPONSE:** Thank you for this suggestion. We have rephrased this sentence as “GKRS was arranged if regrowth of tumor remnant was identified from subsequent postoperative MRI by the senior neurosurgeon” in our revised manuscript.

11. *I’m not sure why there are no line numbers in the paragraphs between lines 155 and 156, but the sentence with the word ‘half-invasion’ needs to be redone.*
    - **RESPONSE:** We are sorry for this mistake and have added the line numbers in that paragraphs. Also, we have corrected this word to ‘half-canal involvement’ in our revised manuscript. Thank you for help.
12. *Line 173: “After then” should be changed*

- **RESPONSE:** Thank you for this suggestion. We have rephrased this sentence as “We checked MRI every 6 to 12 months afterwards to assess tumor control” in our revised manuscript.

13. *Lines 198-204: This paragraph was already stated in the methods section, unclear why it is in the discussion again*

- **RESPONSE:** In this paragraph, we tried to explain that there was no iatrogenic nerve injury and we performed surgery delicately with intraoperative nerve monitoring. We agree with your suggestion that this paragraph was unclear and some redundant sentence. We have revised the text after careful consideration.

14. *Lines 247-250: This sentence is unclear and needs to be revised.*
    - **RESPONSE:** We have rephrased this sentence as “Similar to previous reports, our results support the idea that near-total resection has greater therapeutic outcomes and a lower risk of recurrence. Nonetheless, postoperative follow-ups using serial MRI for the early detection of recurrence are necessary”. Thank you for this suggestion.
15. *Lines 249: Please remove the word ‘alert’*

- **RESPONSE:** Thank you for this suggestion. We have rephrased this sentence mentioned as comment #13.

**REVIEWER 2 COMMENTS:**

1. *The authors provided additional details regarding the statistical methods used. Given the multiple factors that likely affect surgical outcomes - both facial function and tumor control - I would have liked to see multivariate adjustment in the regressions to determine the significance of individual tumor or treatment factors of interest. If this was already done, it is not clear to the reader and should be clarified.*
   - **RESPONSE:** Thank you for this suggestion. Our results of predictive factors for facial palsy had been adjusted by multivariable linear regression initially. We are sorry for vague legends and have clarified that in our manuscript. We supplemented the statistical methods used as following. We performed statistical analyses for tumor control by the chi-square and Fisher’s exact tests due to categorical data.

| **Coefficients^a^** | | | | | | |
| --- | --- | --- | --- | --- | --- | --- |
| Model | | Unstandardized Coefficients | | Standardized Coefficients | t | Sig. |
|  |  | B | Std. Error | Beta |  |  |
| 1 | (Constant) | .577 | .910 |  | .634 | .527 |
|  | AGE | .003 | .009 | .032 | .308 | .759 |
|  | Duration | -.007 | .024 | -.030 | -.285 | .776 |
|  | Tumor size(mm) | .024 | .017 | .147 | 1.460 | .147 |
|  | Pre-OP hearing | .004 | .004 | .122 | 1.120 | .266 |
|  | distance between IAC & jugular bulb mm | -.005 | .045 | -.010 | -.101 | .920 |

| Model | | Unstandardized Coefficients | | Standardized Coefficients | t | Sig. | |
| --- | --- | --- | --- | --- | --- | --- | --- |
|  |  | B | Std. Error | Beta |  |  |  |
|  | Sex | .213 | .243 | .094 | .874 | .384 | |
|  | Tumor type 1.solid 2.heterogenous 3.cyst | .224 | .173 | .137 | 1.296 | .198 | |
|  | IAC involvement 1. 0, 2.1/2, 3.full | .067 | .244 | .029 | .275 | .784 | |
|  | Tumor removal, 1. partial, 2. subtotal, 3. near total, 4. total | -.027 | .188 | -.015 | -.142 | .887 | |
|  | IAC tumor removal 0.no 1. partial removal 2. total removal | .536 | .253 | .232 | 2.114 | .037 | |
|  | Facial numbness (Trigeminal N, involve), 1. yes, 2. no | -.200 | .236 | -.085 | -.849 | .398 | |
|  | Lower cranial nerve involve 1.yes, 2. no | -.106 | .319 | -.035 | -.331 | .741 | |
|  | Pre-OP GKRS 1.yes, 0. no | .288 | .534 | .055 | .540 | .591 | |
|  | Post-OP GKRS 1.yes, 0. no | .614 | .398 | .160 | 1.540 | .127 | |
|  | post-op CSF leakage | -.506 | .364 | -.144 | -1.390 | .168 | |
| a. Dependent Variable: Post-OP facial (6months) | | | | | | |  |

2. *Additionally, I remain concerned that there is adequate duration of follow-up for all patients to draw the stated conclusion regarding tumor control. Consider that in some reports the average time to remnant tumor growth is over 1 year (Kasbekar AV, Adan GH, Beacall A, Youssef AM, Gilkes CE, Lesser TH. Growth Patterns of Residual Tumor in Preoperatively Growing Vestibular Schwannomas. J Neurol Surg B Skull Base. 2018;79(4):319-324. doi:10.1055/s-0037-1607421), yet some patients in the current study have duration of follow-up as short as 6 months. I commend that the authors have acknowledged this as a limitation, yet they make a (admittedly reasonable) conclusion about tumor control regardless. Consider changing the stated conclusions, waiting for longer duration of follow-up, or excluding cases with a shorter duration of follow-up. With regards to postoperative treatment with gamma knife radiosurgery, the authors have clarified that no patients were treated prophylactically. What was the mean growth observed (and ranges of growth) that prompted GKRS in the 10 patients that received it? Specifically, what growth was seen for the patient that received GKRS at 4 months?*
   - **RESPONSE:** Thank you for providing these insights. We arranged MRI scan within three days after surgery to be baseline of postoperative result. The case was treated with GKRS at 4 months because definite enlargement of residual tumor size was noted by the second time of postoperative MRI. We provide the immediate postoperative MRI and post-3 month MRI for supporting the reason we arranged GKRS at postoperative 4^th^ month for the case.

 🡪

According to our medical charts, the case has no further recurrence after GKRS until now. We agree that it was really a limitation of our study with short following time and we mention this in the limitation section of the revised manuscript.

Sincerely,

***Mao-Che Wang***

Corresponding Author

Department of Otorhinolaryngology-Head and Neck Surgery, Taipei Veterans General Hospital, and School of Medicine, National Yang Ming Chiao Tung University, Taipei 112, Taiwan

No. 201, Sec. 2, Shih-Pai Rd.

Tel: +886 2 27857337 ext.151

Fax: + 886 2 28757338

E-mail: [wangmc@vghtpe.gov.tw](mailto:wangmc@vghtpe.gov.tw)
